# A novel *NF1* mutation in a pediatric patient with renal artery aneurysm

**DOI:** 10.1186/s13052-022-01382-8

**Published:** 2022-11-21

**Authors:** Ilenia Chillura, Giulia Angela Restivo, Simonetta Callari, Sabrina Cibella, Maria Michela D’Alessandro, Ciro Corrado, Mario Vallone, Vincenzo Antona, Giovanni Corsello

**Affiliations:** 1 Department of Health Promotion, Mother and Child Care, Internal Medicine and Medical Specialties “G. D’Alessandro”, University of Palermo, Piazza delle Cliniche, 2, 90127 Palermo, Italy; 2 Pediatric Nephrology Unit, ARNAS Ospedali Civico, Di Cristina e Benfratelli, Via dei Benedettini, 1, 90134 Palermo, Italy; 3 Radiology Unit, ARNAS Ospedali Civico, Di Cristina e Benfratelli, Piazza Nicola Leotta, 4, 90127 Palermo, Italy

**Keywords:** Neurofibromatosis type 1, Renal artery aneurysm, Coil embolization, Hypertension

## Abstract

**Background:**

Neurofibromatosis type 1 (NF1) is a neurocutaneous syndrome, due to heterozygous pathogenic variants in *NF1* gene. The main clinical manifestations are multiple café au lait spots, axillary and inguinal freckling, cutaneous and plexiform neurofibromas, optic glioma, Lisch nodules and osseous lesions, such as sphenoid and tibial dysplasia. Vasculopathy is another feature of NF1; it consists of stenosis, aneurysms, and arteriovenous malformations, frequently involving renal arteries.

**Case presentation:**

We report on a 9-year-old girl with a novel mutation in *NF1* gene and renal artery aneurysm, treated by coil embolization and complicated with hypertension.

**Conclusion:**

Vasculopathy is a complication of NF1, affecting from 0.4 to 6.4% of patients with NF1. Among the vascular abnormalities, renal artery aneurysm is a rare manifestation, with only a few cases regarding adult patients and no pediatric reports described in current literature. The finding of a vascular abnormality in a specific site requires the evaluation of the entire vascular system because multiple vessels could be involved at the same time.

## Background

Neurofibromatosis type 1 (NF1) is an autosomal dominant neurocutaneous syndrome due to heterozygous pathogenic variants in *NF1* gene (17q11.2), that is a *de novo* variant in half of affected individuals. NF1 can be diagnosed by physical examination and evaluation of the patient’s family history, according to the National Institutes of Health (NIH) diagnostic criteria, established in 1987 [1]. Recently, an international panel of neurofibromatosis experts was assembled in order to revise the diagnostic criteria for NF1 by incorporating new clinical features and genetic testing [2]. The revised diagnostic criteria are met in an individual who does not have a parent diagnosed with NF1 if two or more of the following are present or in a child of a parent who meets the diagnostic criteria if one or more of the following are present: (a) six or more café-au-lait macules over 5 mm in greatest diameter in prepubertal individuals and over 15 mm in greatest diameter in postpubertal individuals; (b) freckling in the axillary or inguinal region; (c) two or more neurofibromas of any type or one plexiform neurofibroma; (d) optic pathway glioma; (e) two or more iris Lisch nodules identified by slit lamp examination or two or more choroidal abnormalities defined as bright, patchy nodules imaged by optical coherence tomography/near-infrared reflectance imaging; (f) a distinctive osseous lesion such as sphenoid dysplasia, anterolateral bowing of the tibia, or pseudarthrosis of a long bone; (g) a heterozygous pathogenic NF1 variant with a variant allele fraction of 50% in apparently normal tissue such as white blood cells. Other clinical features of NF1 are learning disabilities, central nervous system gliomas, and malignant peripheral nerve sheath tumors.

Vasculopathy is a well-known feature of NF1: renal, aortic, cerebral and other visceral arteries could be involved, both as stenosis and aneurysm [3]. Hypertension is a common finding in patients with NF1. Although essential hypertension is the main cause of hypertension, it can also result from renovascular disease, paragangliomas, pheochromocytoma, and coarctation of the aorta [4].

We now report on a 9-year-old girl with NF1 and renal artery aneurysm (RAA), complicated with hypertension.

## Case presentation

**Fig. 1:**
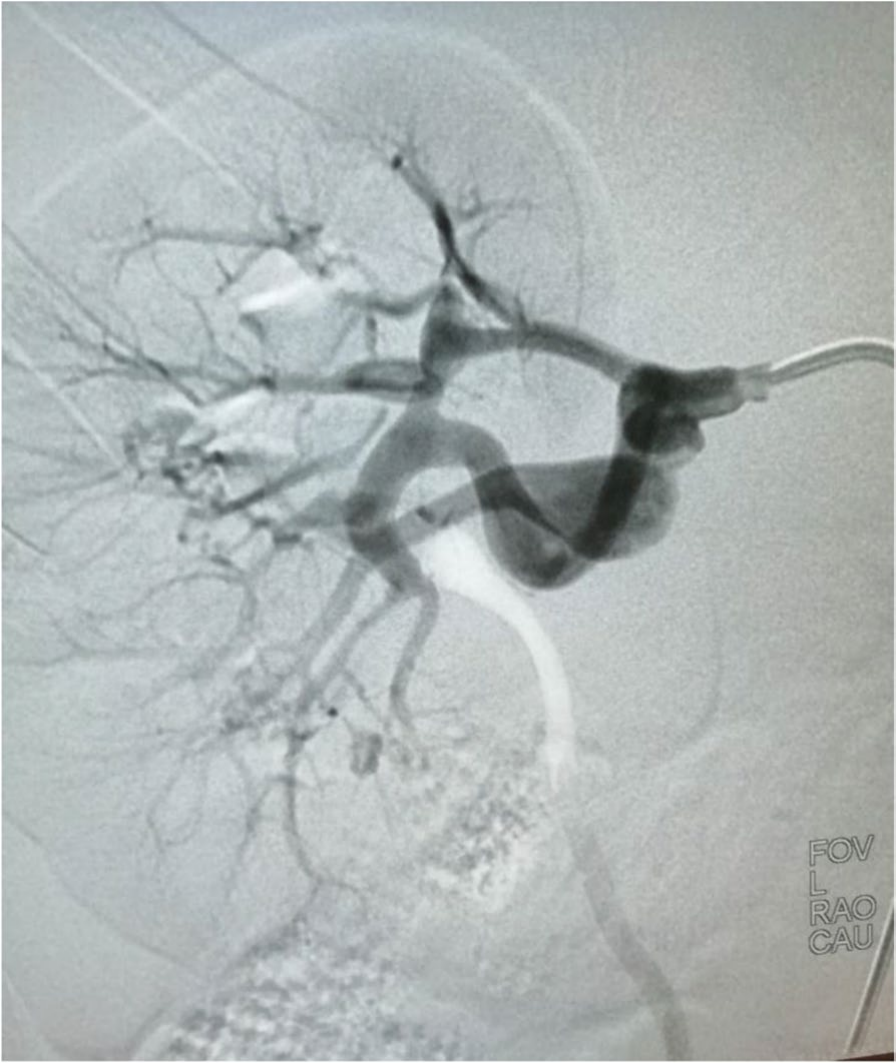
Pre-operative arteriography of the right kidney: a fusiform aneurysm in the upper segmental artery and a dissecting aneurysm in the lower segmental artery were found

**Fig. 2:**
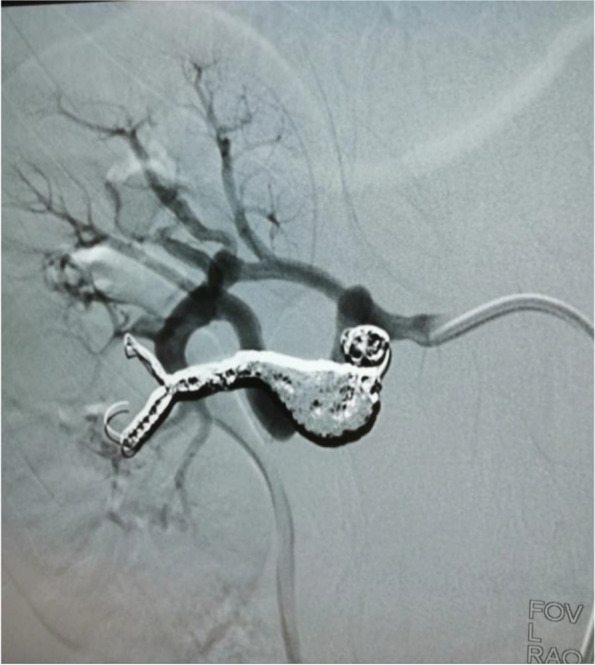
Intra-operative arteriogram of the right kidney: the lower segmental artery was cannulated and the aneurysm sac was embolized with multiple coils

C.C. was born at term of a normal pregnancy, with normal birth growth parameters. Familiar history was unremarkable for genetic disease. No other medical problems were detected in the past medical history. At the age of 9 years, C.C. was conducted for a dermatological consultation due to rosacea. During this evaluation, the physician noticed twenty café-au-lait spots with variable diameter (from 0.5 to 1.5 cm), mostly localized in the trunk and in the superior limbs, and axillary freckling. The girl was referred to a geneticist for the clinical suspicion of NF1, confirmed by next generation sequencing (NGS). A de novo heterozygous c.4206delA mutation was found in NF1 gene, characterized by a single nucleotide deletion resulting in a frameshift in exon 32, that introduces a premature stop codon (p.Ala1424GlnfsTer4). This variant has not been previously reported in the literature or in any public database but it is predicted to be pathogenetic by the prediction tools. A comprehensive eye exam, visual and auditory evoked potentials, and electroencephalogram showed no abnormalities; an abdominal ultrasound with color-doppler revealed the presence of a 10 mm aneurysm in the right renal artery. For this reason, the patient was referred to the Pediatric Nephrology Unit (PNU). The girl was well-appearing, without showing symptoms related to this vasculopathy. On physical examination, she presented multiple café-au-lait macules and no cutaneous neurofibromas were detected. Office blood pressure was 100/60 mmHg and ambulatory blood pressure monitoring (ABPM) demonstrated blood pressure within normal limits according to age, sex and height. Renal computed tomography angiography revealed the presence of a 10,5 mm fusiform aneurysm in the right lower segmental artery, a 6 mm saccular aneurysm in the right upper segmental artery and a 4 mm aneurysm in the right apical segmental artery; brain and chest magnetic resonance angiography did not show other vascular lesions. A renal arteriography confirmed a 7 mm fusiform aneurysm in the right upper segmental artery and a dissecting aneurysm of 15 mm in the right lower segmental artery (Fig. 1). A coil embolization was planned: the right lower segmental artery was cannulated using a microcatheter and the aneurysm sac was embolized with multiple coils (Fig. 2). A new renal arteriography documented a perfusion defect due to the occlusion of the lower segmental artery with a consensual infarcted area. The postoperative course was unremarkable, except for moderate flank pain well-controlled by analgesics, and she was discharged home. Two weeks later, the girl complained of headache and skin flushing: elevated blood pressure (140/100 mmHg) was discovered and she was admitted again to the PNU. During hospitalization, office blood pressure was confirmed elevated (150/100 mmHg) and ABPM showed a stage II hypertension. Renal function was preserved, with no evidence of proteinuria; echocardiogram and ophthalmologic evaluation demonstrated no alterations. Urinary and plasma catecholamines and metanephrines, plasmatic renin activity and aldosterone were within normal range. A renal doppler ultrasound showed no evidence of renal artery stenosis; the lower pole of the right kidney presented an hyperechogenic portion, indicative of ischemic area due to embolization procedure. A diagnosis of secondary hypertension related to partial renal infarction after coil-embolization was made and amlodipine was started; one week after starting therapy, a new ABPM revealed worsening of blood pressure: amlodipine was up-titrated and enalapril was added. Following the therapy adjustment, blood pressure was controlled and the girl was discharged.

## Discussion and conclusions

NF1-vasculopathy includes stenosis, aneurysms and arteriovenous malformations occurring in patients with NF1 [3, 5]. It is an underestimated complication because a diagnosis is usually made only in patients with specific clinical manifestations. The prevalence of vascular lesions in NF1 ranges from 0,4% to 6,4% of all patients [6]. The most common site of involvement is renal artery, which tends to be more frequently stenotic than aneurysmal [7]; in Oderich et al. [3], 76 vascular abnormalities were diagnosed in 31 patients (mean age 38 ± 16 years): only 4% was RAA, while renal artery stenosis accounted for about 12% of all the vascular abnormalities. Other vessels could be affected, such as cerebral, carotid and aortic arteries. Most patients with NF1 vascular abnormalities are asymptomatic. When the renal arteries are involved, hypertension is the most frequent presentation [3]. Both renal artery stenosis and aneurysm can cause renovascular hypertension [8–10]; the pathogenesis of hypertension in renal artery stenosis is well-known (linked to activation of renin-angiotensin-aldosterone system), while the association between RAA and hypertension is not clearly understood, probably related to altered renal flow from kinking or torsion that causes decreased kidney perfusion [11, 12]. Several pathogenetic hypotheses about NF1 vasculopathy were postulated [13]. Some authors suggested that intimal thickening in NF1 vasculopathy is the result of proliferation of Schawann cells within the artery or it depends on compression or invasion by neural tumors. Another theory is related to neurofibromin deficiency; it has been demonstrated by immunohistochemistry that neurofibromin is expressed by endothelial and smooth muscle cells [14] and its deficiency may cause proliferation within vessel wall, a process analogous to that which produces cutaneous neurofibromas [3]. A genotype-phenotype correlation, between a specific pathogenic variant in NF1 gene and vasculopathy, has not yet been found [15]. To our best knowledge, there is no other case of RAA in a child with NF1 reported in current literature. A few cases of RAA in adults NF1 patients have been described, often complicated by hypertension [8–10]; spontaneous rupture without risk factors may occur, with four cases reported [16–19].

For the diagnosis of renal artery abnormalities in patients with NF1, the first-line imaging technique is color-doppler ultrasonography. This procedure is non-invasive and commonly accepted by pediatric patients; it could be used as an instrument of screening in asymptomatic NF1 patients and a follow-up tool in children with any known renal abnormalities. The limitation of color-doppler ultrasonography includes poor cooperation and difficulty in visualizing the entire renal artery due to overlying bowel gas [20]. Other diagnostic modalities are computed tomography, magnetic resonance imaging, and arteriography. In our asymptomatic case, renal color-doppler ultrasound, performed as a screening tool in a patient with NF1, was very useful in the diagnosis of RAA.

As regards treatment, it depends on the type of renal abnormality and the associated symptoms. Firstly, children with hypertension can be managed conservatively through medical therapy; if blood pressure is poorly controlled with anti-hypertensive drugs, surgical intervention is warranted [21]. In the case of RAA, the indications for endovascular treatment (stent-grafting or coil embolization) are size > 2 cm, female gender within childbearing age, symptoms like pain, hematuria, medically refractory hypertension including that associated with functionally important renal artery stenosis, thromboembolism, dissection and rupture [22]. In our case, coil embolization is required because of the presence of a dissecting aneurysm, with an elevated risk of spontaneous rupture. The complications of endovascular procedures include renal infarction, pain, hypertension, and fever. As observed in our case, hypertension occurs after coil embolization, most likely secondary to partial renal infarction. A close surveillance of blood pressure in these patients is needed in order to reveal either the onset of hypertension or the worsening of preexisting hypertension [23].

In conclusion, vasculopathy is a well-known complication in NF1 patients. The finding of a vascular abnormality in a specific site requires the evaluation of the entire vascular system because multiple vessels could be involved at the same time. In our opinion, this case is relevant because among the vascular abnormalities in NF1, RAA is a rare manifestation, with only a few cases regarding adult patients and no pediatric reports described in current literature. Our child was asymptomatic at the diagnosis and the identification of this life-threatening condition was possible thanks to the early starting of the screening program for major NF1 complications. We suggest monitoring the blood pressure of all patients that undergo coil embolization for RAA to facilitate early recognition of hypertension.

## Data Availability

The datasets used and analyzed during the current study are available from the corresponding author on reasonable request.

## Abbreviations

NF1: Neurofibromatosis type 1
RAA: Renal artery aneurysm
NGS: Next generation sequencing
PNU: Pediatric nephrology unit
ABPM: Ambulatory blood pressure monitoring
